# Supplementary material for: Modeling glioblastoma heterogeneity as a dynamic network of cell states
Source: Mol Syst Biol. 2021 Sep 16;17(9):e10105. doi: 10.15252/msb.202010105 (PMC8444284; doi:10.15252/msb.202010105)
Supplement: Supplementary file 6 — Source Data for Figure 5 [file MSB-17-e10105-s004.zip › Figure5A_sourcedata/GSEA_3071/hallmarks_stateIII.GseaPreranked.1623742540963/HALLMARK_COAGULATION.html]

Details for gene set HALLMARK\_COAGULATION[GSEA]

|  || Dataset | state33071 |
| Phenotype | NoPhenotypeAvailable |
| Upregulated in class | na\_neg |
| GeneSet | HALLMARK\_COAGULATION |
| Enrichment Score (ES) | -0.56762254 |
| Normalized Enrichment Score (NES) | -2.7389154 |
| Nominal p-value | 0.0 |
| FDR q-value | 0.0 |
| FWER p-Value | 0.0 |
Table: GSEA Results Summary

  

Fig 1: Enrichment plot: HALLMARK\_COAGULATION      
 Profile of the Running ES Score & Positions of GeneSet Members on the Rank Ordered List

  

| PROBE | GENE SYMBOL | GENE\_TITLE | RANK IN GENE LIST | RANK METRIC SCORE | RUNNING ES | CORE ENRICHMENT || 1 | CSRP1 |  |  | 345 | 0.279 | -0.0735 | No |
| 2 | ARF4 |  |  | 348 | 0.277 | -0.0589 | No |
| 3 | CTSO |  |  | 615 | -0.260 | -0.1131 | No |
| 4 | MAFF |  |  | 735 | -0.266 | -0.1292 | No |
| 5 | TFPI2 |  |  | 1128 | -0.286 | -0.2144 | No |
| 6 | ADAM9 |  |  | 1870 | -0.328 | -0.3869 | No |
| 7 | LAMP2 |  |  | 1956 | -0.333 | -0.3906 | No |
| 8 | CAPN5 |  |  | 1994 | -0.335 | -0.3819 | No |
| 9 | TIMP1 |  |  | 2038 | -0.337 | -0.3747 | No |
| 10 | MMP2 |  |  | 2527 | -0.375 | -0.4796 | No |
| 11 | P2RY1 |  |  | 2613 | -0.383 | -0.4807 | No |
| 12 | MMP14 |  |  | 2811 | -0.400 | -0.5095 | No |
| 13 | CTSB |  |  | 2916 | -0.410 | -0.5140 | No |
| 14 | FURIN |  |  | 2932 | -0.412 | -0.4955 | No |
| 15 | PROS1 |  |  | 3214 | -0.444 | -0.5435 | Yes |
| 16 | HTRA1 |  |  | 3242 | -0.450 | -0.5260 | Yes |
| 17 | C1R |  |  | 3364 | -0.473 | -0.5314 | Yes |
| 18 | PLAU |  |  | 3435 | -0.488 | -0.5229 | Yes |
| 19 | SERPINE1 |  |  | 3474 | -0.499 | -0.5056 | Yes |
| 20 | LRP1 |  |  | 3584 | -0.540 | -0.5042 | Yes |
| 21 | ITGA2 |  |  | 3589 | -0.542 | -0.4758 | Yes |
| 22 | THBS1 |  |  | 3599 | -0.547 | -0.4484 | Yes |
| 23 | PLAT |  |  | 3600 | -0.547 | -0.4187 | Yes |
| 24 | CD9 |  |  | 3634 | -0.567 | -0.3964 | Yes |
| 25 | F2RL2 |  |  | 3645 | -0.574 | -0.3678 | Yes |
| 26 | DUSP6 |  |  | 3648 | -0.577 | -0.3370 | Yes |
| 27 | CFH |  |  | 3653 | -0.582 | -0.3064 | Yes |
| 28 | PRSS23 |  |  | 3658 | -0.584 | -0.2757 | Yes |
| 29 | TIMP3 |  |  | 3755 | -0.677 | -0.2636 | Yes |
| 30 | F3 |  |  | 3764 | -0.693 | -0.2281 | Yes |
| 31 | FBN1 |  |  | 3790 | -0.723 | -0.1953 | Yes |
| 32 | PDGFB |  |  | 3803 | -0.751 | -0.1576 | Yes |
| 33 | SPARC |  |  | 3811 | -0.759 | -0.1182 | Yes |
| 34 | CLU |  |  | 3846 | -0.827 | -0.0821 | Yes |
| 35 | FN1 |  |  | 3850 | -0.842 | -0.0371 | Yes |
| 36 | A2M |  |  | 3890 | -1.053 | 0.0100 | Yes |
Table: GSEA details [plain text format]

  

Fig 2: HALLMARK\_COAGULATION: Random ES distribution      
 Gene set null distribution of ES for **HALLMARK\_COAGULATION**
